# Strategies to prevent *Clostridioides difficile* infections in acute-care hospitals: 2022 Update

**DOI:** 10.1017/ice.2023.18

**Published:** 2023-04

**Authors:** Larry K. Kociolek, Dale N. Gerding, Ruth Carrico, Philip Carling, Curtis J. Donskey, Ghinwa Dumyati, David T. Kuhar, Vivian G. Loo, Lisa L. Maragakis, Monika Pogorzelska-Maziarz, Thomas J. Sandora, David J. Weber, Deborah Yokoe, Erik R. Dubberke

**Affiliations:** 1 Northwestern University Feinberg School of Medicine, Ann & Robert H. Lurie Children’s Hospital of Chicago, Chicago, Illinois, United States; 2 Edward Hines Jr. Veterans’ Affairs (VA) Hospital, Hines, Illinois, United States; 3 Norton Healthcare, Louisville, Kentucky, United States; 4 Boston University School of Medicine, Boston, Massachusetts, United States; 5 Case Western Reserve University School of Medicine, Cleveland VA Medical Center, Cleveland, Ohio, United States; 6 University of Rochester Medical Center, Rochester, New York, United States; 7 Division of Healthcare Quality Promotion, Centers for Disease Control and Prevention, Atlanta, Georgia, United States; 8 McGill University, McGill University Health Centre, Montréal, Québec, Canada; 9 Johns Hopkins University School of Medicine, The Johns Hopkins Hospital, Baltimore, Maryland, United States; 10 Thomas Jefferson University, Philadelphia, Pennsylvania, United States; 11 Boston Children’s Hospital, Harvard Medical School, Boston, Massachusetts, United States; 12 School of Medicine, University of North Carolina, Chapel Hill, North Carolina, United States; 13 University of California San Francisco, UCSF Health-UCSF Medical Center, San Francisco, California, United States and; 14 Washington University School of Medicine, St. Louis, Missouri, United States

## Objective

Previously published guidelines provided comprehensive recommendations for detecting and preventing healthcare-associated infections (HAIs). The intent of this document is to highlight practical recommendations in a concise format designed to assist acute-care hospitals to implement and prioritize their *Clostridioides difficile* infection (CDI) prevention efforts. This document updates the *Strategies to Prevent* Clostridium difficile *Infections in Acute Care Hospitals* published in 2014.^1^ This expert guidance document is sponsored by the Society for Healthcare Epidemiology of America (SHEA) and is the product of a collaborative effort led by SHEA, the Infectious Diseases Society of America (IDSA), the Association for Professionals in Infection Control and Epidemiology (APIC), the American Hospital Association (AHA), and The Joint Commission.

## Summary of major changes

This section lists major changes from the *Strategies to Prevent* Clostridium difficile *Infections in Acute Care Hospitals: 2014 Update*,^1^ including recommendations that have been added, removed, or altered. Recommendations in this document are categorized as “essential practices” that are foundational to all HAI programs in acute-care hospitals (in 2014, these were termed “basic practices”) or “additional approaches” to be considered for use in locations and/or populations within hospitals during outbreaks in addition to full implementation of essential practices (in 2014 these were termed “special approaches”). A complete summary of the recommendations contained in this document is provided in Table 1.

## Essential practices

In the 2014 *Compendium*, encouraging appropriate use of antimicrobials for CDI and other infections was considered an essential practice, but formal adoption of an antimicrobial stewardship program was considered an additional approach. In the 2022 *Compendium*, encouraging appropriate use of antimicrobials by implementing an antimicrobial stewardship program is now recommended as an essential practice. Implementation of diagnostic stewardship practices for appropriate use and interpretation of *C. difficile* testing is a new essential practice recommendation. Assessing adequacy of room cleaning, an additional approach in the 2014 *Compendium*, is now an essential practice recommendation.

## Additional approaches

No new additional approaches have been added.

## Unresolved issues

Identification of asymptomatic carriers of *C. difficile* and then initiating contact precautions, and use of CDI antibiotic prophylaxis for high-risk groups, have been included as unresolved issues.

## Intended use

This document was developed following the process outlined in the *Handbook for SHEA-Sponsored Guidelines and Expert Guidance Documents*.^2^ No guideline or expert guidance document can anticipate all clinical situations, and this document is not meant to be a substitute for individual clinical judgement by qualified professionals. This document focuses on the prevention of CDI in acute-care hospitals. The strategies highlighted may or may not be applicable for other healthcare settings, such as ambulatory settings or long-term or post-acute care facilities. Furthermore, healthcare environments within the hospital may differ (eg, acute-care wards vs intensive care units vs perioperative spaces, etc.), which may affect the feasibility of specific recommendations that should be considered by stakeholders implementing these strategies.

This document is based on a synthesis of evidence, theoretical rationale, current practices, practical considerations, writing group consensus, and consideration of potential harm, where applicable. The evidence-based guidance is limited to strategies that have been demonstrated to reduce clinical infections rather than those that may be associated with reductions in nonclinical outcomes only, such as environmental contamination by *C. difficile*. Additionally, this guidance is focused on prevention of incident CDI, not recurrent CDI. A summary list of recommendations is provided in Table 1.

## Methods

SHEA recruited 3 subject-matter experts in CDI prevention to lead the panel of members representing the *Compendium* partnering organizations: SHEA, IDSA, APIC, AHA, and The Joint Commission, as well as the Centers for Disease Control and Prevention (CDC).

SHEA utilized a consultant medical librarian, who worked with the panel to develop a comprehensive search strategy for PubMed and Embase (January 2012–July 2019; updated to August 2021). Article abstracts were reviewed by panel members in a double-blind fashion using the abstract management software Covidence (Melbourne, Australia). The articles were subsequently reviewed as full text. The *Compendium* Lead Authors group voted to update the literature findings, and the librarian reran the search to update it to August 2021. Panel members reviewed the abstracts of these articles via Covidence and incorporated relevant references.

Recommendations resulting from this literature review process were classified based on the quality of evidence and the balance between desirable and potential undesirable effects of various interventions (Table 2). Panel members met via video conference to discuss literature findings, recommendations, quality of evidence for these recommendations, and classification as essential practices, additional approaches, or unresolved issues. Panel members reviewed and approved the document and its recommendations.

The *Compendium* Expert Panel, made up of members with broad healthcare epidemiology and infection prevention expertise, reviewed the draft manuscript after consensus had been reached by the writing panel. Following review and approval by the expert panel, the 5 partnering organizations, stakeholder organizations, and the CDC reviewed the document. Prior to dissemination, the guidance document was reviewed and approved by the SHEA Guidelines Committee, the IDSA Standards and Practice Guidelines Committee, The Joint Commission, and AHA, and the Boards of SHEA, IDSA, and APIC. All members complied with SHEA and IDSA policies regarding conflict-of-interest disclosure.

## Section 1: Rationale and statements of concern

### Epidemiology of Clostridioides difficile infection (CDI)

1. *C. difficile* is the most common pathogen causing HAIs in the United States (US).^3,4^
2. In the US, *C. difficile* has been classified by the CDC as one of the most urgent antibiotic-resistant public health threats, one that requires “urgent and aggressive action.”^5^ This classification is because of the profound morbidity, mortality, and excess healthcare expenditures associated with CDI.
3. Over the past 20 years, CDI increased among all age groups, including children, but it remains disproportionately higher in the older adult population. Women and individuals identifying as white race also experience higher frequency of CDI.^6^ The proportion of US hospital discharges in which a patient received the *International Classification of Disease, Ninth Revision, Clinical Modification* (ICD-9-CM) discharge diagnosis code for CDI more than doubled between 2000 and 2009.^7^
4. More recently, improvements in those previously described trends were observed. US CDI surveillance performed by the CDC Emerging Infections Program noted that since 2014, CDI incidence has leveled off and is perhaps beginning to decrease.^5,8^ However, this trend was marked by a decrease in healthcare-associated (HA) CDI concomitant with an increase in community-associated (CA) CDI.^8^ CDI with onset outside the hospital now accounts for >50% of US CDI cases. CDI present on admission to the hospital may increase the risk of CDI for other hospitalized patients.^9,10^ Notably, laboratory-identified healthcare-associated CDI decreased during the first year (ie, 2020) of the coronavirus disease 2019 (COVID-19) pandemic.^11,12^
5. CDI incidence increased in the early 2000s concomitant with observations of increased CDI severity.^13-17^ Increases in incidence and severity of CDI were associated with the 027/BI/NAP1 strain of *C. difficile*.^13,17^ However, 027/BI/NAP1 cases has declined significantly in the US^8^, Canada, and Europe.^18^ In the US in 2017, the prevalence of the 027/BI/NAP1 strain was 15% of HA-CDI and 6% of CA-CDI cases. Currently, 027/BI/NAP1 is no longer the predominant US strain. Ribotypes 106, 002, and 014/020 have increased in prevalence over the last several years.^8^

### Burden of outcomes associated with CDI

1. CDI is associated with increased length of hospital stay, costs, morbidity, and mortality in adult and pediatric patients.^19-23^
2. *C. difficile* causes >450,000 infections in the US each year,^6^ including >225,000 cases in hospitalized patients.^5^
3. CDI increases hospital length of stay by 2.8–5.5 days.^20^
4. Approximately 10%–30% of patients experience at least 1 CDI recurrence after an initial episode, and the risk of recurrence increases following each successive recurrence.^24,25^
5. The attributable mortality of CDI is estimated to be 4.5%–5.7% and 6.9%–16.7% during endemic and epidemic periods, respectively.^26^ CDI is associated with 12,000–30,000 US deaths each year.^5,6^
6. Colectomy rates following CDI in hospitalized patients are 0.3%–1.3% and 1.8%–6.2% during endemic and epidemic periods, respectively.^26^
7. Attributable costs of inpatient CDI in 2008 dollars were estimated to be $3,006–$15,397 per episode in adults^20^; more recent US estimates indicate that average CDI-attributable costs exceed $21,000.^27^ Attributable costs are slightly less in children.^23^ US hospital costs for CDI management are estimated at $1.0 billion–$4.9 billion per year.^5,20^
8. Patients with CDI are nearly twice as likely to be discharged to a long-term care facility than propensity score–matched controls.^19^

### Risk factors for CDI

1. Antibiotic exposure is the most important modifiable risk factor for CDI. Virtually every antibiotic has been associated with CDI, even following short antibiotic courses. Antibiotic classes that confer the highest risk of CDI include third- and fourth-generation cephalosporins,^28^ fluoroquinolones,^29^ carbapenems,^28^ and clindamycin.^30^
2. Advanced age and duration of hospitalization are also important CDI risk factors, and these may be proxy measures associated with severity of illness, comorbidities, and antibiotic exposure.^31^
3. Gastric acid suppression, particularly use of proton pump inhibitors, has been recognized as a risk factor for CDI.^32^ The association between CDI and H2-receptor blockers is less established. It remains unclear whether there is an independent association or gastric acid suppression is a proxy for other CDI risk factors,^9,33^ and restriction of gastric acid suppression is not yet established as an effective CDI prevention measure (see Section 4: Unresolved issues, part 8).
4. Other comorbidities^34,35^ that increase CDI risk include cancer chemotherapy, gastrointestinal surgery, enteral feeding tubes, inflammatory bowel disease, and solid organ transplantation.

### Healthcare facility transmission and role of asymptomatic colonization

1. *C. difficile* exposure, and subsequent colonization, are preceding events that are essential to developing CDI. Thus, prevention of exposure and colonization are targets for CDI prevention.
2. *C. difficile* transmission in healthcare facilities likely occurs via contamination of healthcare personnel (HCP) hands,^36^ the care environment,^37-41^ or medical equipment^42^ by *C. difficile* spores.
3. Prevalence of asymptomatic colonization with *C. difficile* during hospitalization is as high as 20%–25% of adults^34^ and children^43^ in some centers. The prevalence of asymptomatic colonization with *C. difficile* at the time of hospital admission is ~8%.^44^
4. *C. difficile* transmission can originate both from patients with CDI and those with asymptomatic colonization.^45-47^ Studies^48,49^ demonstrating that symptomatic patients contribute to only a minority of HA-CDI cases suggest that other reservoirs for transmission may be underrecognized, including patients with asymptomatic colonization.

## Section 2: Background on detection of CDI

### Surveillance definitions for CDI

1. Various surveillance definitions are used for healthcare-associated CDI, and standardization in CDI surveillance definitions is needed. The following information focuses on the definitions for CDI surveillance in the United States^9,34,50,51^ and Europe.^52^
  - A clinical CDI case is defined as a case of clinically significant diarrhea or toxic megacolon without other known etiology that meets 1 or more of the following criteria: (1) the stool sample yields a positive result for a laboratory assay for *C. difficile* toxin A and/or B, or a toxin-producing *C. difficile* organism is detected in the stool sample by culture or other means; (2) pseudomembranous colitis is seen on endoscopic examination or surgery; and/or (3) pseudomembranous colitis is seen on histopathological examination. Large-scale surveillance efforts may rely solely on laboratory evidence of CDI (ie, LabID events) (see Section 2: Surveillance definitions for CDI, part 1e) because surveillance for clinical history may not be feasible or reliable across all healthcare facilities.
  - The definition of clinically significant diarrhea has not been validated either for stool quality or quantity. In terms of stool consistency, diarrheal stool may be operationally defined as stool that is unformed and adheres to shape of its container. The Bristol Stool Scale may assist in scoring stool quality (ie, unformed stools defined as Bristol score 5–7). In terms of stool quantity, diarrhea is defined at least 3 or >3 diarrheal bowel movements within 24 hours.
  - HCP should document frequency and consistency of stools in the medical record.
  - Recent outbreaks of severe CDI indicate that it is not always possible to wait 24–48 hours before determining whether a patient has clinically significant diarrhea; therefore, diarrhea plus abdominal cramping has also been used to satisfy criteria for clinically significant diarrhea.^53,54^ Conversely, it is normal for some patients to have 3 or more bowel movements per day. However, these bowel movements are usually formed. Therefore, it is not possible to provide strict criteria for clinically significant diarrhea that can be applied to all patients. In general, clinically significant diarrhea in the context of CDI should consist of a sustained change in bowel movement consistency and/or frequency with or without abdominal cramping in a patient without other identified causes.
  - Several CDI definitions have been proposed, and the most commonly used surveillance definitions are listed in Table 3. Healthcare facilities should track at least healthcare facility-onset CDI (Table 3).^55^
    - Hospitals in the US typically apply the National Healthcare Safety Network (NHSN) LabID event definitions to CDI,^56^ as reporting CDI incidence through NHSN is required for certain Centers for Medicare & Medicaid Services (CMS) payment programs for acute-care facilities. This reporting focuses on positive laboratory tests in relation to hospital admission and does not consider the presence or timing of onset of symptoms. Healthcare facility–onset CDI is defined as having a positive nucleic acid amplification test (NAAT) or toxin (based on the result of the last test performed if a multistep algorithm is done) ≥4 days after healthcare facility admission, with the day of admission counted as day 1. An event may be identified as ‘recurrent’ when there is a previous event at the same facility in the previous 56 days. If the event is the first for that patient at the facility or day 57 or longer from previous event, the event is identified as an incident of CDI. An equation is used to determine the predicted number of hospital-onset CDI cases for a hospital based on the hospital characteristics, type of *C. difficile* testing done, and number of people admitted with community-onset CDI. The standardized infection ratio (SIR) is then calculated by dividing the number of observed healthcare facility–onset CDI cases by the number of predicted healthcare facility–onset CDI cases.
    - Because the result of the last test performed in a multistep testing algorithm dictates whether a case is reportable to the NHSN, the pattern of results of tests performed in a different order can significantly impact the SIR. For example, a glutamate dehydrogenase (GDH)–positive, toxin enzyme immunoassay (EIA)–negative result that is followed by a positive NAAT is considered reportable to NHSN as a CDI case, but a NAAT-positive result followed by a negative-toxin EIA is not reportable to the NHSN as a CDI case. This discordance represents a weakness in the surveillance definition because clinical management is not dictated solely by the result of the last test performed. However, data from the CDC (not yet published) suggest that >75% of patients are treated for CDI despite having a negative toxin EIA following a positive NAAT even though data suggest that treatment may not be necessary.^12,13^ Failing to report this volume of clinical CDI cases based on order of test performance biases SIR measurements and interfacility comparisons.
    - The NHSN is updating the healthcare facility-onset CDI surveillance definition to incorporate antibiotic treatment in addition to test results (ie, healthcare facility-onset, treated CDI [HT-CDI]).
      - Data have demonstrated the existence of patients with a positive test for *C. difficile* who do not meet the current NHSN definition for healthcare facility-onset CDI but who ultimately received treatment for CDI, suggesting that they were determined to have clinically significant CDI and should likely be considered a CDI case for surveillance purposes.
      - The updated definition is still undergoing validation, but it will involve a combination of any positive test for *C. difficile* plus initiation of antibiotics specifically for treatment of CDI.
        - For the most likely proposed definition, a case of HT-CDI will be defined as any positive test for *C. difficile* on or after hospital day 4 from admission, and in whom ≥5 days of CDI treatment were given, and treatment was started within 2 calendar days of the positive *C. difficile* test. If a patient is discharged or transferred before receiving 5 days of treatment, any treatment will count.
        - Data submitted to meet this metric are expected to be available in 2023.
        - Until the HT-CDI definition and corresponding SIR adjustment can be validated, the current healthcare facility-onset CDI definition will continue to be used as the outcome measure for CDI surveillance and SIR reporting for the purposes of CMS payment programs.
  - Surveillance for CDI is limited by variation in patient selection for testing, lower sensitivity of toxin EIA, lower specificity of NAAT, and prolonged turnaround time for the cell-culture cytotoxicity assay as well as stool culture for toxigenic *C. difficile*.^53,57,58^ Lack of culture-based methods for routine diagnosis also limits the availability of strains for molecular typing, but at least 1 commercially available NAAT for *C. difficile* will provide a presumptive identification of the BI/NAP1/027 strain.

### Surveillance methods for CDI

1. Conducting CDI surveillance to determine CDI rates provides a measure to determine the burden of CDI at a healthcare facility. These data are also utilized to assess the efficacy of interventions to prevent CDI. When reported back to HCP and hospital administrators, CDI rates can be applied as a tool to improve adherence to CDI preventive measures.
  - When conducting CDI surveillance, healthcare facilities can use traditional infection surveillance reporting or laboratory-based reporting.
    - Traditional reporting involves chart review to determine the date of symptom onset and whether the patient meets the surveillance definition for CDI. Potential cases are typically identified by a stool laboratory test positive for toxigenic *C. difficile* and/or its toxins.
    - Laboratory-based reporting also utilizes positive tests to identify cases, but chart review is not performed. Rather, it is assumed that all positive tests represent patients with CDI, and the date of stool collection is used as a proxy for date of symptom onset.
  - Comparisons between the methods of surveillance have been performed, and the 2 methods typically have good concordance in correctly categorizing CDI cases into the proper surveillance definition.^59,60^
    - Although there are concerns that laboratory-based surveillance is less accurate and more likely to incorrectly classify community-onset CDI cases as healthcare-facility onset, excellent sensitivity and specificity of an electronic surveillance algorithm has been demonstrated.^59^ Even with the potential for some misclassification, the time saved by laboratory-based surveillance is often determined to outweigh the risk.^59,60^ In addition, identification of misclassification is an opportunity for improvement.
    - Rapid identification and implementation of contact precautions for patients with CDI is paramount to prevent *C. difficile* transmission. Patients with community-onset CDI who are not identified until it is classified as health-care facility-onset CDI represent delays in CDI diagnosis and initiation of contact precautions.
2. Surveillance can be performed on specific wards or units and/or an entire healthcare facility level.
3. Infection prevention and control programs should have a system in place for reviewing results of positive *C. difficile* tests in patients included in their CDI surveillance plan to ensure accurate and complete case ascertainment. The healthcare facility-onset CDI rate can be expressed as the number of CDI case patients per 10,000 patient days. The calculation of this rate is (number of case patients ÷ the number of inpatient days per reporting period) × 10,000 = rate per 10,000 inpatient days.
4. Outbreaks and hyperendemic rates can occur at the ward level.
  - An outbreak can be defined as an increase in CDI in time and/or space believed to be greater than that expected by chance alone for a given healthcare facility or ward.
  - A hyperendemic rate can be defined as a persistently elevated CDI rate compared to past rates or compared to other similar healthcare facilities and/or wards.

### Identification of patients with CDI and appropriate test utilization

1. Background:
  - Positive results of diarrheal stool tests for toxigenic *C. difficile* (ie, NAAT) or its toxins (ie, EIA) are the most common methods to identify patients with CDI.^34,50,51,61^ A minority of patients are diagnosed by visualizing pseudomembranes at endoscopy and/or by histopathology without stool testing.^61^ NAATs, which detect toxigenic *C. difficile*, are extraordinarily sensitive but do not reliably differentiate *C. difficile* colonization and infection. Toxin EIAs, which detect *C. difficile* toxins, are less sensitive than NAATs but have greater clinical predictive value for CDI.^62,63^
  - These distinctions between test types are important because *C. difficile* colonization can occur in up to 20%–25% of children and adults over the course of their hospitalization and is more likely to occur with prolonged and/or repeated hospitalizations. Thus, the specific test used and the scenarios during which patients are tested will affect the clinical predictive value of the test and the likelihood of misdiagnosis of *C. difficile* colonization as CDI. Several diagnostic stewardship strategies are effective for reducing misdiagnosis of CDI in individuals who are colonized with *C. difficile* (Section 2: Identification of patients and appropriate test utilization, part 1b).
  - The impact of CDI misdiagnosis: Frequent misdiagnosis of *C. difficile* colonization as CDI falsely increases institutional CDI rates, which may be publicly reported. Misdiagnosis impairs reliable interfacility comparisons of CDI rates and increases inappropriate use of antibiotics for CDI, which may result in increased healthcare costs, risk of antibiotic-related adverse events, antimicrobial resistance, and prolonged use of contact precautions (ie, isolation).^64^
2. Potential strategies for improving test utilization:
  - Institutions should establish criteria for CDI test collection, processing, and test interpretation. This is important irrespective of test type but is particularly important when NAATs are used either as a standalone test or multi-step testing algorithm.^34^ If a multistep algorithm is used, hospitals should develop clinical practice guidance for the treatment of patients who are toxin EIA positive versus those positive only by NAAT. HCP should receive education about the availability and use of that clinical practice guideline.
  - Testing criteria may include several factors, including the presence of diarrhea, recent CDI testing history, and the presence of factors that increase likelihood of other non-CDI diarrheal etiologies.^34^ Evidence-based testing strategies include the following:
    - When diagnosing CDI, only test patients with clinically significant diarrhea for *C. difficile* or its toxins. Clinically significant diarrhea is defined as 3 or more unexplained and new-onset, unformed stools in the 24-hour period prior to testing. Unexplained diarrhea implies lack of an alternative explanation, but HCP should be aware that CDI and other potential alternative explanations may not be mutually exclusive (eg, patients on laxatives or who recently started enteral feeds can also have CDI). Effort should be taken to discern diarrhea chronology and associated symptoms to discern CDI from alternative explanations to guide testing decisions.
      - Testing of those without diarrhea should not be part of routine clinical practice (see Section 4: Essential practices, part 2).
      - For patients without clinically significant diarrhea, testing should only be pursued if other CDI signs or symptoms are present that may reduce stool output, such as ileus or toxic megacolon.
    - Prior to testing for *C. difficile* in patients with new-onset diarrhea, thoughtful consideration should be given to other potential infectious or noninfectious diarrhea etiologies. This includes current use of medications that result in diarrhea, such as laxatives. In some circumstances, it may be reasonable to hold laxatives to observe for resolution of diarrhea before sending *C. difficile* testing.
    - Repeated testing for *C. difficile* should not be performed during the same episode of diarrhea (ie, within 7 days).
    - Because of the high prevalence of asymptomatic colonization of toxigenic *C. difficile* among infants and children aged <2 years, testing for CDI is not advised in children aged <1 year, and testing for CDI in children aged 1–2 years should be deferred until other more likely infectious or noninfectious diarrhea etiologies have been excluded.
    - Most patients who are clinically cured with treatment will continue to have toxigenic *C. difficile* in their stool for multiple weeks or longer, which is not an indication of treatment failure. Therefore, test of cure should not be conducted, even if a patient is being transferred to another healthcare facility. Facilities should not require repeat *C. difficile* testing to confirm “clearance” of the organism prior to accepting a patient for interfacility transfer.
  - Care must be given to balance diagnostic stewardship strategies with also avoiding underutilization of testing that could potentially lead to missed CDI cases. Frequently missed opportunities to test adults with new onset of diarrhea has been reported at some hospitals, but its impact on patient outcomes and/or *C. difficile* transmission is unknown.^65^
  - Several diagnostic stewardship strategies have safely and successfully reduced misdiagnosis of *C. difficile* colonization as CDI, with resultant reduction in hospital CDI incidence, CDI antibiotic use, and healthcare charges. These strategies include those below. The comparative effectiveness of these interventions is unknown, although leveraging electronic resources has the advantage of better ensuring consistency in the diagnostic stewardship approach. Hospitals can consider implementation of 1 or more based on cost, resources, and feasibility:
    - Clinical microbiology assessment of stool consistency by various methods and rejection of formed stools for testing
    - Education of ordering providers^66,67^ and bedside nurses^68,69^ about appropriate CDI testing decisions
    - Audit and feedback of CDI testing orders regarding appropriateness of testing^70^
    - Real-time computerized provider order entry alerts and decision support tools^67,71,72^
    - Electronic medical record tracking of clinically significant diarrhea and laxative use at time of *C. difficile* testing ordering.^73^

## Section 3: Background on prevention of CDI

### Summary of existing guidelines and recommendations

1. Limitations of existing guidance. Published guidelines on the management of CDI have expanded in recent years, but only some address CDI prevention.^34,52,74,75^ Most published studies of CDI prevention are single-center studies with a quasi-experimental (ie, before-and-after or pre- and postintervention) or other observational nonexperimental study design, often performed in response to outbreaks or elevated CDI rates. Often, several concomitant interventions are performed, making it difficult to determine the relative importance of one intervention compared to another. Before-and-after studies are also limited by time-related biases that are difficult to adjust for in the absence of a control group or properly conducted analyses such as interrupted time-series analysis.^76,77^ However, several studies have utilized these techniques, in some cases leading to meta-analyses.
2. Unique microbiologic characteristics of *C. difficile. C. difficile* shares many common epidemiologic characteristics with other antimicrobial-resistant gram-positive organisms such as methicillin-resistant *Staphylococcus aureus* (MRSA) and vancomycin-resistant *Enterococcus* (VRE). Both the skin and the environment of colonized patients become contaminated, and HCP hands may become contaminated by touching the environment or the patient.^78-81^ The major difference among these 3 organisms is that only *C. difficile* forms spores. The formation of spores poses unique challenges for hand hygiene and environmental disinfection practices because *C. difficile* spores are resistant to the bactericidal effects of alcohol and commonly used hospital disinfectants (eg, phenolics and quaternary ammonium compounds).
3. Strategies focused on unique microbiologic characteristics inconsistently result in CDI prevention. Although alcohol-based hand rub is ineffective at removing or disinfecting *C. difficile* spores in controlled laboratory experiments, no clinical study has demonstrated an increase in CDI with the use of these products or a decrease in CDI with soap and water.^82-88^ Conversely, several of the studies did identify decreases in MRSA^83-85,88^ or VRE^84^ associated with the use of alcohol-based hand rub. Similarly, use of sporicidal methods to disinfect the environment outside outbreak settings has not consistently demonstrated a reduction in CDI with these methods.^89-91^ These data indicate that although the environment can be an important source of *C. difficile*, indirect transmission by HCP may be the predominant route by which patients acquire *C. difficile*.
4. Strategies to prevent CDI (discussed in Section 4) in acute-care settings are generally categorized as follows:
  - Approaches to minimize *C. difficile* exposure and colonization through transmission by HCP (eg, hand hygiene and contact precautions)
  - Approaches to minimize *C. difficile* exposure and colonization through transmission from the environment (eg, cleaning and disinfection of environment and shared medical devices)
  - Approaches to reduce the risk of *C. difficile* colonization and CDI if the organism is encountered by the patient (eg, antimicrobial stewardship).

### Infrastructure requirements

1. Trained infection preventionists. Infection preventionists must have knowledge about risk factors and methods to prevent CDI. They must also be trained in how to categorize CDI cases using surveillance definitions and how to calculate CDI rates.
2. Methods to systematically identify patients with CDI. Infection preventionists must be able to identify patients with CDI as soon as possible after they are diagnosed, in order to ensure patients are placed in contact precautions in a timely fashion. These data can also be used to calculate CDI rates.
3. Ability to place patients with CDI on contact precautions:
  - Contact precautions require the ability to place patients in a private room (preferably) or cohort patients with CDI.
  - Place materials necessary for adherence with contact precautions (eg, gowns and gloves) in an easily accessible space outside of the patient room. Hand washing sinks should be readily accessible to HCP following doffing of personal protective equipment and/or care of patients with suspected or confirmed CDI.
  - Place a sign indicating that the patient is on contact precautions outside the patient’s room. This sign should be in English as well as any other language that is commonly spoken in the community or among HCP.
  - Patients with stool incontinence should preferentially be placed in private rooms. If private rooms are unavailable, use of a dedicated commode or bathroom is recommended.
  - If it is necessary to cohort patients, place colonized or infected patients in cohorts with the same organism(s). For example, do not cohort patients with CDI who are discordant on VRE or MRSA colonization status.
  - Dedicated equipment (eg, stethoscopes) should be readily available for HCP. If dedicated equipment is not available, responsibility for who will clean and disinfect equipment, when it will be cleaned and disinfected, and how it will be cleaned and disinfected must be clearly stated.
  - Have systems in place to facilitate communication among infection prevention and control, admitting, nursing, and environmental service departments, and develop contingency plans for limited bed availability conditions.
4. An antimicrobial stewardship program is an important part of many quality and safety metrics, including CDI prevention (see Section 4: Essential practices, part 1). A more detailed description of antimicrobial stewardship program infrastructure has been published by Barlam et al.^92^
5. Provide educational materials for patients, family members, and HCP that include explanations of CDI, why contact precautions are necessary, and the importance of hand hygiene.
6. Provide adequate resources and training for environmental service personnel to ensure proper cleaning of rooms.

## Section 4: Recommended strategies for CDI prevention

Recommendations are categorized as either (1) essential practices that should be adopted by all acute-care hospitals or (2) additional approaches that can be considered for use in locations and/or populations within hospitals when CDI incidence is not controlled by use of essential practices. Essential practices include recommendations in which the potential to impact CDI risk clearly outweighs the potential for undesirable effects. Hospitals can prioritize their efforts by initially focusing on implementing the prevention approaches listed as essential practices. If CDI surveillance or other risk assessments suggest ongoing opportunities for improvement, hospitals should then consider adopting some or all of the additional approaches. These can be implemented in specific locations or patient populations, or they can be implemented hospital-wide, depending on outcome data, risk assessment, and/or local requirements. After literature review and discussion by the author panel, each recommendation was assigned a quality of evidence (Table 2).

### Essential practices for preventing CDI recommended for all acute-care hospitals

1. Encourage appropriate use of antimicrobials through implementation of an antimicrobial stewardship program. (Quality of evidence: MODERATE)
  - Ensure appropriate use of antimicrobials for CDI treatment: HCP should work with their antimicrobial stewardship program to ensure that patients with CDI are receiving appropriate severity-based treatment based on current guidance,^34,75^ which may improve clinical outcomes of CDI in these patients. Antimicrobial stewardship guidance should be paired with diagnostic stewardship guidance to ensure appropriate interpretation of *C. difficile* diagnostic tests and confirm that CDI therapy is needed (see Section 4: Essential practices, part 2). These efforts are particularly important considering the proposed incorporation of CDI treatment in an updated CDI surveillance definition (ie, HT-CDI).
  - Ensure the appropriate use of non–CDI-treatment antimicrobials.^34^
    - The major risk factor for hospitalized patients to develop CDI is antecedent antimicrobial exposure.^93,94^ Although any systemic antibiotic may increase the risk of CDI, fluoroquinolones, third- and fourth-generation cephalosporins, carbapenems, and clindamycin are associated with the highest risk of CDI.^34^ Encouraging appropriate antimicrobial use has been associated with reductions in CDI incidence in both endemic and outbreak settings.^95-98^ Two meta-analyses suggest that implementation of an antimicrobial stewardship program reduces CDI incidence by 30%–50%.^99,100^
    - Appropriate antimicrobial use includes avoiding antimicrobial exposure if the patient does not have a condition for which antimicrobials are indicated, deescalating antibiotic therapy when feasible, and selecting antimicrobials associated with a lower risk of CDI when possible.^101^
    - Antimicrobial stewardship programs that are restrictive (ie, that require approval prior to antibiotic administration) are likely more effective for reducing CDI than programs that are persuasive (eg, that audit antimicrobial use and give direct feedback to the HCP).^100^
    - The effectiveness of antimicrobial stewardship programs for reducing CDI incidence has been reported both for programs targeting antibiotic use generally^99,100^ and for those that target high-risk antibiotics specifically,^97^ such as fluoroquinolones^102-105^ and cephalosporins.^104,105^
    - Restriction of specific high-risk antimicrobials (eg, clindamycin, cephalosporins, and fluoroquinolones) may be a targeted approach that can be utilized specifically during CDI outbreaks or based on local CDI epidemiology.^106-108^ For example, a decline in the incidence of the fluoroquinolone-resistant 027/BI/NAP1 strain has been associated with targeted efforts to reduce fluoroquinolone use.^109^
    - Appropriate use of non–CDI-treatment antimicrobials may be particularly important for patients with history of CDI and/or *C. difficile* colonization.
2. Implement diagnostic stewardship practices for ensuring appropriate use and interpretation of *C. difficile* testing. (Quality of evidence: LOW)
  - Hospital infection prevention and control programs should work with their clinical microbiology laboratory to develop pre-agreed criteria for *C. difficile* testing, particularly if NAATs are used either as a standalone test or part of a mul-tistep testing algorithm (see Section 2: Identification of patients and appropriate test utilization).^34^ Inclusion of the antibiotic stewardship program in these discussions may assist in optimizing CDI treatment decisions based on test results.
  - At minimum, *C. difficile* testing should be avoided in patients without clinically significant diarrhea, in those who have been tested in the prior 7 days, and in children aged <1 year.^34^ Additional action may be taken to reduce testing in individuals with diarrhea from a more likely etiology such as recent laxative use or initiation of enteral tube feeding.
  - Ordering providers^66,67^ and bedside nurses^68,69^ should receive education about appropriate use and interpretation of *C. difficile* testing. Bedside nurses frequently identify patients with diarrhea before the treating physician does and some hospitals have standing orders or nurse-driven protocols to test patients with diarrhea for *C. difficile*. In these circumstances, education of optimal *C. difficile* testing practices should be performed before implementing nurse-driven protocols, and education should be continually reinforced among nursing staff.
  - If feasible, the electronic medical record system should be leveraged to provide computerized provider order entry support and/or monitoring for clinical testing criteria (see Section 2: Identification of patients and appropriate test utilization).^67,71-73^
3. Use contact precautions for infected patients, single-patient room preferred. (Quality of evidence: LOW for hand hygiene; MODERATE for gloves; LOW for gowns; LOW for single-patient room)
  - Perform hand hygiene based on CDC or World Health Organization (WHO) guidelines before and after entering the room (ie, immediately before donning and after removing personal protective equipment). Using soap and water prior to the use of alcohol-based hand rubs is recommended as an additional strategy (see Section 4: Additional approaches, part 2).
  - Place patients with CDI on contact precautions to help reduce patient-to-patient spread of the organism.
    - Place patients in private rooms when available.
    - Don gown and gloves upon entry to the patient’s room. Gloves should be changed immediately if visibly soiled, after touching or handling surfaces or materials contaminated with feces, or after moving from a dirty area of patient care (eg, high-touch surface or likely contaminated area of the body of a patient) to a clean area (eg, patient wound or indwelling device).
    - Make dedicated patient care equipment (eg, stethoscopes) readily available.
      1. Use dedicated equipment whenever possible.
      2. If equipment is shared between patients (eg, glucometers), do not bring the equipment into the patient room if possible.
      3. Clean and disinfect the piece of equipment immediately after use. Identify who will clean and disinfect, and how to clean/disinfect, each piece of shared equipment.
    - Remove gown and gloves prior to exiting the room and then perform hand hygiene.
  - Cohorting of patients with CDI^110^ is acceptable when single private rooms are not available.
    - An intensive care unit (ICU)–based study found admission to a room of a patient with CDI to be a risk factor for CDI, but 90% of patients who developed CDI did not have this risk factor.^111^ Other studies that have examined sharing a room with a patient diagnosed with CDI or being admitted to a room after a patient with CDI was discharged from that room, have not found these exposures to be risk factors for CDI.^79,93,112,113^
    - Place patients with stool incontinence preferentially in private rooms.
    - Do not cohort patients who are discordant for other epidemiologically important organisms (eg, VRE or MRSA).
    - Remove gowns and gloves and perform hand hygiene when moving from one patient to the other.
  - Ensure that adequate supplies for contact precautions are readily available.
    - Clinical and hospital supply chain management leaders together are responsible for ensuring that necessary barrier equipment supplies (eg, gowns, gloves), dedicated equipment, and hand hygiene products are readily available.
    - Assign responsibility for monitoring the availability and restocking of supplies to specific HCP.
  - Criteria for discontinuing contact precautions:
    - The CDC currently recommends^114^ contact precautions for patients with CDI for at least 48 hours after diarrhea has resolved. This is the recommendation for patients who have diarrhea and are positive by NAAT, irrespective of EIA result (ie, even if patient is *C. difficile* positive but suspected to be colonized and have an alternate cause of diarrhea). Contact precautions can potentially be prolonged up to the duration of hospitalization, and this is considered an additional approach (see Section 4: Additional approaches, part 3).
    - After resolution of symptoms, patients with CDI can continue to shed *C. difficile* in stool and contaminate the environment.^115^ In addition, these patients are at high risk for recurrent CDI after treatment is stopped. Currently, data do not exist to support extending contact precautions as a measure to decrease CDI incidence. Therefore, extending contact precautions until discharge for all patients with CDI remains an additional approach.
    - Area of controversy: Asymptomatic colonized patients who have not had CDI can shed *C. difficile* spores, but the number of spores and degree of contamination is not as great as for patients with active CDI.^113^ Identification of these patients and initiation of contact precautions may prevent *C. difficile* transmission but this issue remains unresolved (see Section 4: Unresolved issues).
4. Adequately clean and disinfect equipment and the environment of patients with CDI. (Quality of evidence: LOW for equipment; LOW for environment)
  - *C. difficile* spores contaminate the environment in which patients are housed and the equipment used to care for them.^116^ The environment includes the following:
    - Furnishings in the room such as over-bed tables, bedrails, furniture, sinks, floors, commodes, and toilets
    - Patient care equipment that directly touches patients, such as thermometers, stethoscopes, and blood pressure cuffs
    - Surfaces touched by HCP and/or patients such as door knobs and intravenous infusion pumps
  - *C. difficile* may contaminate surfaces outside patient rooms, but the frequency of contamination and the number of spores is much lower than are typically present on surfaces inside the rooms of patients with CDI.^117,118^
  - Contaminated surfaces and equipment are potential reservoirs for transmission of *C. difficile*.
    - Data are conflicting as to whether environmental inactivation of spores is necessary to prevent *C. difficile* transmission, especially in an endemic setting.
    - As an additional approach, facilities should consider using a 1:10 dilution of sodium hypochlorite (household bleach) or other product with the US Environmental Protection Agency (EPA)–approved claim for *C. difficile* sporicidal activity^119^ to disinfect the environment in outbreak and hyperendemic settings in conjunction with other infection prevention and control measures (see Section 4: Essential practices, part 4). The solution should have a contact time that meets the manufacturers’ recommendations for *C. difficile* spores.
    - Touchless disinfection technologies is an unresolved issue (see the discussion in Section 4: Unresolved issues, part 2).
  - Develop and implement protocols for disinfection of equipment and the environment.
    - On a routine basis, assess adherence to protocols and the adequacy of cleaning and disinfection.^120^
    - Assess the adequacy of cleaning and disinfection practices before changing to a new cleaning product (eg, bleach). If cleaning and disinfection practices are not adequate, address this before changing products (see Section 4: Essential practices, part 5).
    - Ensure that patient care equipment (eg, wall mounted sphygmomanometers) and electronic equipment (eg, computers) that remain in the patient room are cleaned/disinfected.
    - Educate environmental service personnel on proper cleaning and disinfection technique frequently. The frequency of education may need to be increased if personnel turnover is high. Ensure that education is provided in the native language of personnel.
  - Dedicate noncritical patient-care items, such as blood pressure cuffs, stethoscopes, and thermometers, to a single patient with *C. difficile*. When this is not possible, ensure adequate cleaning and disinfection of shared items between patient encounters. Ensure that manufacturers’ recommendations for contact time of disinfectants are followed.
5. Assess the adequacy of room cleaning. (Quality of evidence: LOW)
  - Work with the environmental services team to establish a process for assessing adequacy of room cleaning at a frequency that is feasible for the team.
  - The process should focus on reviewing and improving cleaning and disinfection techniques. Important issues to address include proper dilution of cleaning and disinfection products, adequacy of cleaning and disinfection technique, cleaning “high-touch” surfaces, frequency of changing rags and mop water, and moving from “clean” areas to “dirty” areas.
    - Create a unit-specific check list based on cleaning protocols and perform observations to monitor cleaning practice.
    - Some studies have demonstrated improved cleaning and disinfection through use of fluorescent markers to monitor thoroughness of cleaning or ATP bioluminescence to measure organic material on surface.^41,121^ However, in another study, fluorescent markers to provide monitoring and feedback of thoroughness of room cleaning did not lead to adequate reductions in *C. difficile* spores from the environment and other enhanced disinfection methods were required.^42^
    - Environmental cultures for *C. difficile* are difficult to perform and may require media not commercially available, and therefore are not routinely recommended.^122^
  - Consider environmental decontamination with an EPA-approved sporicidal agent if room cleaning and disinfection is deemed to be adequate but there is ongoing *C. difficile* transmission (see Section 4: Essential practices, part 4b).
6. Implement a laboratory-based alert system to provide immediate notification to infection preventionists and clinical personnel about newly diagnosed patients with CDI. (Quality of evidence: LOW)
  - To place patients with CDI on contact precautions in a timely manner, it is important that an alert system be developed between the laboratory and both infection preventionists and the clinical personnel caring for the patient. This alert system should promptly notify infection preventionists and clinical personnel when a patient is newly diagnosed with CDI.
  - This information can be transmitted using a variety of methods. Some options include fax alerts, phone call and pager alerts, or automated secure electronic alerts. The alert system should not rely solely on passive communications that do not push notifications to those HCP who need to act on the information immediately, such as faxes or emails to infrequently monitored inboxes.
  - Alert patient care areas of positive test results immediately so that these patients can be placed on contact precautions as soon as possible. Clear protocols indicating who is responsible for reporting a positive test result to the patient care location and who can implement and remove patients from contact precautions should be available.
  - When a patient has CDI (or another current or prior infection requiring isolation), communicate the CDI and isolation status when transferring the patient to another healthcare facility so appropriate precautions can be implemented at the accepting facility.
7. Conduct CDI surveillance and analyze and report CDI data. (Quality of evidence: LOW)
  - At a minimum, calculate healthcare facility-onset CDI rates at the organizational level and consider specifically calculating CDI rates by unit or ward (Table 3).^55^
  - Provide CDI rates and CDI prevention process measures to key stakeholders including senior leadership, physicians, nursing staff, and other clinicians.
  - Provide the process and outcome measures outlined in the “Performance Measures (see Section 5) to appropriate hospital staff and administrators on a regular basis. The frequency at which these data are provided will depend upon the hospital’s existing reporting structure and the type of data collected. These data can be added to routine quality assessment and performance improvement reports.
8. Educate HCP, environmental service personnel, and hospital administration about CDI (Quality of evidence: LOW), including risk factors, routes of transmission, local CDI epidemiology, patient outcomes, and treatment and prevention measures.
9. Educate patients and their families about CDI as appropriate. (Quality of evidence: LOW)
  - Although often not considered part of a program to reduce transmission of CDI and/or multidrug-resistant organisms, proper education may help to alleviate patient and family fears regarding being placed in contact precautions.^36^
  - Include information about anticipated questions: general information about CDI, colonization versus infection, the hospital’s CDI prevention program, the components of and rationale for contact precautions, the risk of transmission to family and visitors while in the hospital and after discharge, and importance of hand hygiene by staff, patients, and visitors. Helpful materials might include patient education sheets in appropriate language(s), the use of patient education channels, websites, or DVDs.
10. Measure compliance with the CDC or WHO hand hygiene and contact precautions recommendations. (Quality of evidence: LOW)
  - Patient-to-patient transmission of *C. difficile* is thought to occur primarily through transient contamination of the hands of HCP with spores.
  - Glove use when caring for patients with CDI or touching surfaces in their rooms has been shown to be effective at preventing the transmission of *C. difficile*.
  - Hand hygiene practices in compliance with CDC or WHO guidelines may be important to *C. difficile* control and prevention. Evidence-based recommendations for implementation and assessment of hand hygiene programs in healthcare settings have been published.^37^
  - Area of controversy: Although gloving is clearly a priority when caring for patients with CDI, the best hand hygiene practice after removing gloves is controversial. There are concerns regarding reliance on alcohol-based hand rub because alcohol is not sporicidal. Several controlled studies have found alcohol-based hand rub to be ineffective at removing or inactivating *C. difficile* spores from the hands of volunteers contaminated with a known number of spores compared to hand washing.^38,39^ Notably, one study did find a reduction of spores from the palmar surface of the hand with the alcohol-based hand rub,^38^ and another recent publication found most hand wash products produced a <1-log_10_ reduction in spores despite a 60-second hand wash (30-second wash and 30-second rinse).^40^ When considering whether or not to promote hand washing over alcohol-based hand rub after caring for a patient with CDI, consider that contamination of hands is less common when gloves are worn for the patient encounter.^79^ And, as previously stated, several clinical studies have not found an increase in CDI with alcohol-based hand rub, but several did find reductions in MRSA and/or VRE.^82-88,123^

### Additional approaches for preventing CDI

In addition to ensuring compliance with the essential recommendations, additional approaches may be added to the CDI prevention program. Additional approaches are (1) approaches in which the intervention is likely to reduce CDI risk but where there is concern about the risks for undesirable outcomes; (2) approaches in which the quality of evidence is relatively low; and (3) approaches in which evidence supports the impact of the intervention in select settings (eg, during outbreaks) or for select patient populations.

When CDI incidence remains higher than the institution’s goal, a CDI risk assessment should be performed. Components of this risk assessment should include, but not necessarily be limited to, determining the location or unit of new CDI cases within the affected area (ie, repeated cases in the same room or cases distributed across multiple sites), the adequacy of contact precautions compliance, the adequacy of hand hygiene, and the adequacy of environmental and equipment cleaning. Additionally, there may be opportunities for improved antibiotic and/or diagnostic test utilization. Meetings with leadership and HCP in the affected area should be conducted to identify potential opportunities to improve the CDI prevention plan. Contact the laboratory that performs the *C. difficile* assay(s) to determine if there have been any changes in assay(s) or assay performance.^124^

1. Intensify the assessment of compliance with process measures. (Quality of evidence: LOW)
  - Contact precautions: gowns and gloves should be worn by all HCP who enter the rooms of patients on contact precautions.
  - Hand hygiene: hand hygiene should be performed at least on entry and exit from patient rooms. When hand washing is performed, determine whether proper technique is being used. If hand hygiene compliance or technique are not adequate, conduct interventions to improve hand hygiene compliance and technique.
  - Assess opportunities for improved antibiotic and/or diagnostic test utilization with improved compliance with and/or using additional antibiotic or diagnostic stewardship approaches (see Section 4: Essential practices, parts 1 and 2).
2. As the preferred method, perform hand hygiene with soap and water following care of or interacting with the healthcare environment of a patient with CDI. (Quality of evidence: LOW)
  - When considering a CDI-specific hand hygiene measure, the priority should be to ensure adherence to donning gloves and proper technique when doffing to minimize the risk for self-contamination.
  - Ideally, after removing gloves, hand hygiene is performed before exiting the patient room when feasible.
  - Ensure proper hand hygiene technique when using soap and water.
  - Be aware that hand hygiene adherence may decrease when soap and water is the preferred method.^37^
    - Gloves are effective at preventing *C. difficile* contamination of hands.^79^
    - Hand washing may remove <1-log_10_ of spores, even with a 60-second hand wash.^40^
    - Alcohol-based hand rub is superior to hand washing for non–spore-forming organisms (eg, MRSA). Using alcohol-based hand rub following soap and water may enhance hand hygiene effectiveness.
    - Reductions in CDI have not been observed with hand washing only using soap and water.^82-88^
3. Place patients with diarrhea on contact precautions while *C. difficile* testing is pending. (Quality of evidence: LOW)
  - Patients with new-onset diarrhea that is unexplained should be placed on contact precautions when diarrhea is recognized. Employ measures, particularly the use of gowns and gloves and disinfection of shared medical equipment (see Section 4: Essential practices, part 3). Contact precautions should be initiated as soon as diarrhea symptoms are recognized because this is the period of greatest *C. difficile* shedding and contamination.^115^
  - Availability of private rooms or ability to cohort patients in nonprivate rooms before a CDI diagnosis is made may be a challenge for some hospitals. Because only a small minority of individuals with diarrhea in a hospital will have *C. difficile*, initiation of full contact precautions in a private or cohort room prior to test results may lead to unnecessary cohort restrictions or patient transfers to private rooms.
    - The decision to place a patient on contact precautions in a private or cohort room while testing is pending can be based on several factors, including likelihood that the patient will transmit *C. difficile*, turnaround time of CDI test results, and impact of contact precautions on hospital bed management.
    - The pretest probability of CDI is increased by certain clinical factors, such as recent history of CDI, high-risk antibiotics (see Section 4: Essential practices, part 1), and/or signs or symptoms of fulminant CDI, such as toxic megacolon. Other factors such as high-volume stool output, stool incontinence, and/or presence of an ostomy may increase the likelihood of CDI transmission. These factors can be considered when deciding on pre-emptive contact precautions while *C. difficile* testing is pending.
    - Movement to a private or cohorted room while awaiting test results is recommended at centers where *C. difficile* test turnaround time is >12–24 hours.
  - If *C. difficile* testing is negative, and another infectious etiology that requires contact precautions is not suspected, contact precautions can be discontinued based on test type and clinical suspicion for CDI.
    - Because of its high negative predictive value, patients with a negative NAAT can be removed from contact precautions.
    - Some hospitals diagnose CDI using only toxin EIAs, for which concerns persist regarding suboptimal sensitivity compared with NAATs. When only toxin EIAs are used, clinical suspicion for CDI should outweigh a negative test result. If there is high pretest probability of CDI, the patient should remain on contact precautions.
4. Prolong the duration of contact precautions after the patient becomes asymptomatic until hospital discharge. (Quality of evidence: LOW)
  - For patients with CDI, CDC currently recommends^114^ contact precautions for at least 48 hours after diarrhea resolves. However, some hospitals may choose to extend contact precautions for the duration of hospitalization even if symptoms have resolved. This is the recommendation for patients who have diarrhea and are positive by NAAT, irrespective of EIA result (ie, even if patient is *C. difficile* positive but is suspected to be colonized and to have an alternate cause of diarrhea).
  - Facilities must balance potential reduction in *C. difficile* transmission with individual patient risk of isolation related to contact precautions, which may include falls and socioemotional stress that can lead to symptoms such as behavior changes, anxiety, depression, and anger.
5. Use an EPA-approved sporicidal disinfectant, such as diluted (1:10) sodium hypochlorite, for environmental cleaning and disinfection. Implement a system to coordinate with environmental services if it is determined that sodium hypochlorite is needed for environmental disinfection. (Quality of evidence: LOW)
  - Sporicidal disinfectants registered with the EPA, including sodium hypochlorite, can be found in EPA List K.^119^
  - Data have not been consistent regarding the ability of sporicidal disinfectants, including diluted sodium hypochlorite, to control CDI through environmental decontamination. However, a beneficial effect has been reported when bleach has been used in outbreak or hyperendemic settings, typically in conjunction with other enhanced CDI control measures.^125-128^
  - When an EPA-approved sporicidal disinfectant is instituted for environmental decontamination, it is necessary to coordinate activities with environmental services.
    - Clinical staff, infection prevention and control staff, and environmental service staff need to determine the location, type, and frequency of sporicidal disinfectant use.
      1. Room type: Use for all patient rooms, only rooms of patients with CDI, and/or outside of patient rooms and in common spaces.
      2. Cleaning timing and frequency: Use for daily cleaning and/or terminal cleaning only when the patient is discharged or transferred. Daily disinfection of touchable surfaces in rooms of patients with CDI and MRSA has been shown to reduce acquisition of the pathogens on investigators’ hands after contact with surfaces and to decrease contamination of the hands of the HCP caring for the patients.^129^
  - When diluted (1:10) sodium hypochlorite is used, it is important to address the following issues:
    - Avoid toxicity to patients and staff and damage to equipment and the environment from bleach use. Sodium hypochlorite can be corrosive and irritating to patients, environmental service personnel, and other HCP.
    - Prior to application of diluted sodium hypochlorite, surfaces need to be cleaned to remove organic matter.
    - Either use a freshly prepared diluted sodium hypochlorite solution or store appropriately.^130^
  - When a sporicidal method will be used only in rooms of patients with CDI, a system will need to be created to identify these patients to environmental service staff.
  - Touchless disinfection technologies remains an unresolved issue (see Section 4: Unresolved issues, part 2).

### Unresolved issues

Several unresolved issues regarding CDI prevention remain. Strategies identified as unresolved were characterized as such for 1 or more reasons: (1) little to no data supporting effectiveness for preventing CDI in hospitals; or (2) some data support implementation but there are concerns of potential patient adverse events with use and there are cost and/or logistical or operational challenges associated with implementation. As a result, implementation of the recommendations beyond the essential practices to prevent CDI should be individualized at each healthcare facility. In a “tiered” approach, recommendations are instituted individually or in groups; additional “tiers” are added if CDI rates do not improve, and essential practices are implemented as the first tier. Additional strategies in subsequent tiers should be prioritized based on the findings of the CDI risk assessment. Some centers with ongoing elevated CDI incidence after implementing essential and additional strategies may choose to adopt 1 or more unresolved strategies after a thorough ongoing risk assessment is performed.

1. Identification of asymptomatic carriers of toxigenic *C. difficile* using rectal or perirectal swabs and NAAT testing and placing those who are positive on contact precautions.
  - Selection of patients for carrier detection has been done in a variety of ways: all emergency department admissions,^131^ all new admissions to specific high-risk wards,^132^ and all admitted patients who had been previously hospitalized within 2 months, and/or had a past *C. difficile* positive test, and/or were in a long-term care facility in the prior 6 months.^133^
  - If a patient has diarrhea but is thought to be a carrier of *C. difficile* with an alternative diarrheal etiology (eg, NAAT positive, toxin EIA negative), contact precautions should still be employed.
  - For asymptomatic carriers, all components of contact precautions for *C. difficile* carriers may not be required. One study demonstrated reduced HA-CDI using a modified contact precautions approach for carriers. Gloves, soap-and-water hand hygiene, dedicated toilet or commode and medical equipment, and a privacy curtain were used, but modified contact precautions did not require gown use or private rooms.^131^ On the other hand, in a recent, large, cluster-randomized trial, universal gown and glove use in intensive care units failed to prevent CDI acquisition.^134^
  - If carrier detection is employed, the number of patient days on contact precautions will increase significantly compared with facilities that do not identify carriers. Hospital CDI transmission may decrease over time with this approach, and the proportion of patients on contact precautions for CDI may decrease over time.^135^ Because asymptomatic colonization is much more frequent than healthcare facility–onset CDI, the decrease in total number of patient days on contact precautions will depend on local healthcare facility-onset CDI rates.
  - Antibiotics to eradicate the carrier state are generally not indicated and represent an unresolved issue.
  - In an asymptomatic patient who has recently recovered from CDI, a repeated test of cure is not indicated. A positive test at the end of therapy does not predict who will develop a recurrence or relapse.^136^
  - Screening for asymptomatic colonization may have additional disadvantages in pediatric settings. *C. difficile* colonization rates are higher in the first 3 years of life and can exceed 40% in infants aged <1 year. Universal screening for colonization in children may therefore detect high numbers of carriers. Placing all colonized children on contact precautions may have negative effects on cohorting, throughput, and workflows, and it may result in dissatisfaction for families if movement outside the room or use of common spaces is restricted. In pediatric settings where this approach is being considered, screening could be limited to children aged >3 years (in whom colonization rates are similar to those of adults).
2. Implementation of touchless disinfection technologies:
  - Several touchless disinfection products are commercially available. In general, these products use ultraviolet light (UV-C) or vaporized hydrogen peroxide (VHP) to disinfect the environment.^42,89,137^ These devices inactivate *C. difficile* spores, and several studies have found them to be effective at reducing cultivatable *C. difficile* from patient rooms.^42,89,137^ Although sporicidal activity can be achieved without requiring a person to wipe down a surface, the use of these devices does not preclude the need to manually clean soiled surfaces.^42,89^
  - Multiple single-center quasi-experimental studies, summarized in a recent meta-analysis,^138^ have shown variable results with touchless disinfection systems, depending on the type of system used (VHP vs UV-C), baseline CDI incidence and the type of chemical disinfection used along with the touchless system (eg, bleach or standard quaternary ammonium). In this meta-analysis, the study quality was low, but 4 of 6 studies that assessed addition of UV-C to bleach cleaning demonstrated a decrease in CDI incidence.
  - In a cluster-randomized, multicenter, crossover study,^139^ UV-C/bleach was compared to bleach alone and was used to disinfect rooms at the time a patient with CDI was discharged. The incidence of CDI among subsequent patients admitted to those rooms did not differ, suggesting little benefit of UV-C with bleach, unlike prior single-center studies. However, also in this study, UV-C was assessed in addition to standard quaternary ammonium clean of rooms occupied by patients with other common healthcare-associated pathogens. In a secondary analysis,^140^ hospital-wide decreases in CDI incidence were observed following implementation of UV-C with standard quaternary ammonium (or with bleach for rooms previously occupied by a patient with *C. difficile*). The results from the primary and secondary analyses of this study suggest that the sporicidal effects of UV-C may be beneficial when added to standard disinfection processes to minimize transmission from patients not previously known to be shedding *C. difficile*.
3. Use of probiotics as primary prophylaxis:
  - Numerous single-center studies have shown variable results for probiotic prophylaxis. Studies have varied considerably in terms of study design and size; type, dose, and duration of probiotic; and baseline risk for CDI.
  - Limitations of prior studies. Two meta-analyses indicated that probiotics may be effective as primary prophylaxis against CDI.^141,142^ A concern with these meta-analyses is that the studies with the greatest weight had extremely high incidences of CDI in the placebo groups (7%, 24%, and 40%). The incidence of CDI in high-risk patients without contraindications to probiotics is typically ≤3%.^143,144^ The high incidence of CDI in the placebo group has the potential to bias the findings to favor the probiotics. For example, a recent, large, randomized controlled trial of probiotic versus placebo with a more typical CDI incidence in the placebo arm (1.2%) failed to demonstrate a reduction in CDI with the use of a probiotic.^145^ Many hospitalized patients may have relative contraindications to probiotics (eg, central venous catheter, immune compromise, ICU admission, gut mucosal barrier compromise) which may place them at increased risk of infection (eg, bacteremia or fungemia) caused by the probiotic strain(s).^146,147^
  - A more recent meta-analysis included nearly 10,000 patients in 39 studies.^148^ This study stratified effectiveness of probiotics by baseline risk of CDI. A benefit of probiotics (relative risk 0.30; 95% confidence interval, 0.21–42) was only demonstrated in studies performed in a population of study participants with a baseline risk of CDI >5%. This finding suggests that prescribing probiotics should only be considered for primary prevention of CDI in those with CDI risk >5% and for those to whom it is safe to administer.
  - Barriers to implementation of probiotics are numerous.
    - The optimal probiotic formulation, dose, duration, and timing of initiation (eg, upon CDI diagnosis or near completion of CDI therapy) are unknown. However, a recent meta-analysis suggests that short-term use of *S. boulardii* or *Lactobacillus acidophilus* plus *L. casei* at a dose of 10–50 billion CFU per day is best supported by the limited available evidence.^148^
    - Probiotics are regulated as nutritional supplements in a manner less rigorous than drug products regulated by the US Food and Drug Administration. Quality control in terms of precision of reported dose and probiotic variability may be lacking for some products.
4. CDI antibiotic prophylaxis for certain very high-risk patients who are receiving systemic antibiotics:
  - Recent systematic reviews and meta-analyses^149,150^ are conflicted about the benefit of oral vancomycin prophylaxis for the primary prevention of CDI.
  - Findings from a small, single-center, HCP-blinded, randomized controlled trial suggest that prophylaxis may be beneficial.^151^ This study assessed the effectiveness of vancomycin as the primary prevention for CDI in adults with multiple CDI risk factors. Vancomycin was specifically compared to no prophylaxis in certain high-risk hospitalized patients receiving systemic antibiotics. Vancomycin 125 mg by mouth once daily was given while receiving systemic antibiotics until 5 days after discontinuing systemic antibiotics. High-risk patients were those at least 60 years of age who were hospitalized and received antibiotics within 30 days prior to the index admission. None (0%) of 50 patients who received vancomycin prophylaxis experienced CDI compared with 6 (12%) of 50 patients who did not receive prophylaxis (*P* = .03).
  - A randomized controlled trial of fidaxomicin prophylaxis in adults undergoing hematopoietic stem-cell transplant was associated with reduced risk of confirmed *C. difficile*–associated diarrhea after transplant.^152^
  - Because of the relatively limited data for both effectiveness and risks (eg, antimicrobial resistance), antibiotic primary prophylaxis for CDI should only be considered for carefully selected patients at very high risk for CDI and only when CDI incidence is elevated despite implementation of other prevention measures.
  - Although beyond the scope of this document, increasingly available literature supports use of antibiotics for secondary prophylaxis for CDI in patients with recent CDI to prevent CDI recurrence in some adults^149,150,153,154^ and children.^155^ Effectiveness estimates are conflicting and additional research is needed. The reader is referred to these studies for further information.
5. Use of gowns and gloves by family members and other visitors:
  - The benefit of requiring family members and other visitors to wear gowns and gloves to prevent *C. difficile* transmission is unknown.^156^ The risk that family members and other visitors will transmit *C. difficile* between patients is likely to be related to the degree of contact the visitor has with the patient and the patient’s environment, whether the visitor performs hand hygiene, and the degree of interaction the visitor has with other patients.
  - At a minimum, family members and other visitors should be instructed to perform hand hygiene whenever entering or leaving the patient’s room. If family members do not wear gowns and gloves, they should be educated about and instructed to use proper hand-washing technique prior to leaving the patient’s room.
  - Compliance with infection prevention measures by visitors is particularly important for pediatric patients because the visitor is nearly always a parent or other primary caregiver who has close contact with the child and participates in routine care activities, such as diapering and toileting.
6. Use of admission-based alert systems that notify infection preventionists and clinical personnel about readmitted or transferred patients with a history of CDI:
  - This information can be integrated into a computerized database used during admission and registration or into a separate electronic or paper-based database.
    - If an alert system is implemented, readmitted patients with a history of CDI should be placed on contact precautions only if they have symptoms consistent with CDI on admission. Asymptomatic patients with a history of CDI do not require contact precautions.
    - The duration that the alert should remain active is unknown. Nearly all cases of recurrent CDI occur within 90 days of the last episode. Therefore, it is reasonable to eliminate the alert after 90 days from the last episode of CDI. However, healthcare facilities may not be aware of recurrent episodes of CDI that are diagnosed and managed in outpatient settings, so an arbitrary cutoff based on the last known episode of CDI may inadvertently remove patients with ongoing recurrent CDI.
7. Ongoing assessment of CDI knowledge and intensified CDI education among HCP. Although re-education of staff about CDI during periods of elevated CDI rates often occurs, it is unknown if this is an effective strategy for CDI prevention.
8. Restriction of gastric acid suppressants. Gastric acid suppressive medications, particularly proton pump inhibitors, increase risk of CDI by ~20%.^32,34,157^ Although it is reasonable to discontinue gastric acid suppressants in patients when no longer needed, whether programs restricting their use effectively prevent CDI remains unclear.

## Section 5: Performance measures

### Internal reporting

These performance measures are intended to support internal hospital quality-improvement efforts and do not necessarily address external reporting needs. The process and outcome measures suggested here (Table 4) are derived from published guidelines, other relevant literature, and the opinions of the authors. Report process and outcome measures to senior hospital leadership, nursing leadership, and clinicians who care for patients at risk for CDI.

1. Process measures: Perform ongoing measurement of recommended CDI prevention practices to permit risk assessment of CDI.
  - Compliance with hand hygiene guidelines.
    - Preferred measure for hand hygiene compliance
      1. Numerator: number of observed proper hand hygiene episodes performed by HCP.
      2. Denominator: total number of observed opportunities for hand hygiene.
      3. Multiply by 100 so that the measure is expressed as a percentage.
    - If hand hygiene with soap and water is the preferred method of hand hygiene when caring for patients with CDI, also assess proper hand washing technique.
      1. Numerator: number of proper hand washing episodes with proper technique.
      2. Denominator: total number of hand washing episodes observed.
      3. Multiply by 100 so that the measure is expressed as a percentage.
  - Compliance with contact precautions:
    - Preferred measure of contact precautions compliance
      1. Numerator: number of observed patient care episodes in which contact precautions are appropriately implemented.
      2. Denominator: number of observed patient care episodes in which contact precautions are indicated.
      3. Multiply by 100 so that the measure is expressed as a percentage
  - Compliance with environmental cleaning and disinfection:
    - One specific measure of compliance for use in all hospitals is not realistic. Many hospitals use checklists, environmental rounds, fluorescent markers, and/or ATP bioluminescence to assess the cleaning and disinfection process and cleanliness of equipment and the environment (see Section 4: Essential practices, part 5).
2. Outcome measures: Perform ongoing measurement of the incidence density of CDI to permit longitudinal assessment of outcomes related to the processes of care. CDI rates are calculated as follows:
  - Numerator: number of CDI cases in the population being monitored (specific cases included in the numerator depends on the definition used) (Table 3).
  - Denominator: total number of patient days in the population being monitored.
  - Multiply by 10,000 so that measure is expressed as number of cases per 10,000 patient days.

### External reporting

There are many challenges in providing useful information to consumers and other stakeholders while preventing unintended adverse consequences of public reporting of HAIs.^158^ Recommendations for public reporting of HAIs have been provided by the Hospital Infection Control Practices Advisory Committee (HICPAC), the Healthcare-Associated Infection Working Group of the Joint Public Policy Committee, and the National Quality Forum.^158,159^

1. State and federal requirements:
  - The Centers for Medicare & Medicaid Services (CMS) began requiring acute-care hospitals participating in their Inpatient Prospective Payment System to report laboratory-identified CDI using the NHSN in January 2013.
  - For information on local requirements, check with your state or local health department.
2. External quality initiatives. Hospitals that participate in external quality initiatives must collect and report the data if required by the initiative.

## Section 6: Implementation strategies

Prevention of CDI relies on the integration of best practices in a culture that supports their implementation. Accountability is one translational link to prevent practices from being performed in an inconsistent and fragmented way, beginning with senior leaders who provide the imperative for HAI prevention and allocate adequate resources, including necessary personnel (clinical and nonclinical), education, and equipment.

The 4 Es—engage, educate, execute, and evaluate—is one example of a widely used model in the US.^127^ The 4 Es model involves summarizing evidence, identifying local barriers, measuring performance, and ensuring that patients receive the intervention.^160,161^ This is done by addressing knowledge, critical thinking, behavior, psychomotor skills, attitudes, and beliefs of members of the healthcare team. Effective strategies to address CDI within healthcare settings are provided in this section.

### Engagement

A broad scope of involvement of multidisciplinary HCP, with engagement of team members who work in the prevention and care of patients with CDI, helps address the complexities involved in implementing a specific CDI control plan based on a risk assessment.^127^ Identify and engage a multidisciplinary team as the initial step in implementing a CDI prevention plan:

1. Involve representation from senior leadership, unit-level leadership, individual HCP, laboratory personnel, pharmacy, environmental services, materials management, and information technology.
2. Establish goals and embed accountability in the process.

### Education

1. Provide education to HCP, environmental services personnel, executive level leadership, and others that includes at least the following elements: risk factors for CDI, transmission, local epidemiology, patient outcomes, treatment, hand hygiene, contact precautions, management of MDROs, and individual job responsibilities.^37,162^
2. Provide information in the native language of the HCP whenever possible.
3. Identify and implement methods for education and training that provide immersive experiences to enhance critical thinking and decision-making skills (eg, simulations).^163^
4. Provide education to patients and their families regarding CDI that at least includes the following elements:
  - The importance of properly performing hand hygiene^164^
  - General information about CDI, including risk for recurrent CDI, and the difference between colonization and infection
  - How the facility works to prevent CDI (eg, relevant elements of its CDI prevention program)
  - Components of and rationale for contact precautions
  - Risks of transmission to family and visitors while in the hospital and after discharge.

### Execution

1. Initiate a CDI prevention program:
  - Perform a CDI risk assessment as a basis for a comprehensive and multidisciplinary intervention.^127^
  - Define local CDI epidemiology.
  - Identify the following locations:
    - High-risk wards
    - Wards with a high incidence of healthcare facility-onset CDI. Determine whether healthcare facility–onset CDI cases are sporadic or occur repeatedly in the same room(s).^165^
      1. If sporadic, this suggests patient-to-patient transmission from HCP or traveling fomites.
      2. If repeatedly occurring in the same room, this suggests transmission from contaminated environment.
  - Initiate the prevention program in which there is a high concentration of patients at risk for CDI, such as an ICU or an oncology ward. Pilot test the intervention in 1 patient care location to assess efficacy.
  - Identify opportunities to improve the following elements:
    - The system for identifying patients with CDI.
    - The process for placing patients with CDI in contact precaution rooms that minimizes problems for family members, visitors, and HCP.
    - Compliance with hand hygiene, contact precautions, and environmental cleaning.
  - Standardize care processes and practices using bundles, checklists, protocols, and guidelines.^125,127^
  - Empower staff to report process defects to appropriate HCP as a means of identifying barriers and facilitating rapid intervention.
  - Obtain support of the hospital administration and local physician and nursing leadership prior to starting the program.
  - Assign accountability for adherence to specific departments or functions.^127^
  - Create redundancy in the system by incorporating use of visual cues as reminders and assistance to recall:
    - Indicators in the electronic health record that the patient is in contact precautions,
    - Paper medical records
    - Signage on the door to the patient room
  - Replicate the CDI infection prevention and control program in other patient care areas when it is determined that the systems developed are effective.

### Evaluation

1. Conduct performance monitoring to determine whether the intervention is effective by using the following:
  - Process measures (ie, did you successfully implement your intervention?)
  - Outcome measures (ie, how well did the intervention achieve the desired outcome?)
2. Measure both process and outcomes on a regular basis.
3. Provide feedback to staff.
4. Provide monitoring data in various formats so it can be posted and broadly distributed.
5. Incorporate monitoring data into unit-based and department-based measurements so trending over time can be evaluated.^125,127^
6. Provide feedback to all levels of personnel regarding process and outcomes, eg, via committee reports and facility newsletters.
7. Format feedback so respective patient-care areas and individual departments can use data for comparative and goal-setting purposes.
8. Use feedback to determine specific interventions or improvements for targeted focus.^161^

## Figures and Tables

**Table 1.** Summary of Recommendations to Prevent *Clostridioides difficile* Infection (CDI)

Essential Practices
1. Encourage appropriate use of antimicrobials through implementation of an antimicrobial stewardship program. (Quality of evidence: MODERATE)
a. Ensure appropriate use of antimicrobials for CDI treatment.
b. Ensure appropriate use of non–CDI-treatment antimicrobials.
2. Implement diagnostic stewardship practices for ensuring appropriate use and interpretation of *C. difficile* testing. (Quality of evidence: LOW)
a.Hospital infection prevention and control programs should work with their clinical microbiology laboratory to develop pre-agreed criteria for *C. difficile* testing, particularly if NAATs are used either as a standalone test or part of a multi-step testing algorithm.
b. At minimum, *C. difficile* testing should be avoided in patients without clinically significant diarrhea, in those who have been tested in the prior 7 days, and in children aged <1 year.
c. Ordering providers and bedside nurses should receive education about appropriate use and interpretation of *C. difficile* testing.
d. If feasible, the electronic medical record system should be leveraged to provide computerized provider order entry support and/or monitoring for clinical testing criteria.
3. Use contact precautions for infected patients, single-patient room preferred. (Quality of evidence: LOW for hand hygiene; MODERATE for gloves; LOW for gowns; LOW for single-patient room)
a.Perform hand hygiene based on CDC or WHO guidelines before and after entering the room (ie, immediately before donning and after removing personal protective equipment).
b. Place patients with CDI on contact precautions to help reduce patient-to-patient spread of the organism.
c. Cohorting of patients with CDI is acceptable when single private rooms are not available.
d. Ensure that adequate supplies for contact precautions are readily available.
e. Follow appropriate criteria for discontinuing contact precautions.
4. Adequately clean and disinfect equipment and the environment of patients with CDI. (Quality of evidence: LOW for equipment; LOW for environment)
a.*C. difficile* spores contaminate the environment in which patients are housed and the equipment used to care for them.
b. Contaminated surfaces and equipment are potential reservoirs for transmission of *C. difficile*.
c. Develop and implement protocols for disinfection of equipment and the environment.
d. Dedicate noncritical patient care items, such as blood pressure cuffs, stethoscopes, and thermometers, to a single patient with *C. difficile*.
5. Assess the adequacy of room cleaning. (Quality of evidence: LOW)
a.Work with the environmental services team to establish a process for assessing adequacy of room cleaning at a frequency that is feasible for the team.
b. The process should focus on reviewing and improving cleaning/disinfection techniques. Important issues to address include proper dilution of cleaning/disinfection products, adequacy of cleaning/disinfection technique, cleaning “high-touch” surfaces, frequency of changing rags/mop water, and moving from “clean” areas to “dirty” areas.
c. Consider environmental decontamination with an EPA-approved sporicidal agent if room cleaning/disinfection is deemed to be adequate but there is ongoing *C. difficile* transmission.
6. Implement a laboratory-based alert system to provide immediate notification to infection preventionists and clinical personnel about newly diagnosed patients with CDI. (Quality of evidence: LOW)
a.To place patients with CDI on contact precautions in a timely manner, it is important that an alert system be developed between the laboratory and both infection preventionists and the clinical personnel caring for the patient.
b. This information can be transmitted using a variety of methods. Options that push notifications to those HCP who need to act on the information immediately are preferred, such as phone call and pager alerts or automated secure electronic alerts. The alert system should not rely solely on passive communications that may delay receipt of results, such as faxes or emails to infrequently monitored inboxes.
c. Alert patient care areas of positive test results immediately so that these patients can be placed on contact precautions as soon as possible.
d. When a patient has CDI (or another current or prior infection requiring isolation), communicate the CDI/isolation status when transferring the patient to another healthcare facility so appropriate precautions can be implemented at the accepting facility.
7. Conduct CDI surveillance and analyze and report CDI data. (Quality of evidence: LOW)
a.At a minimum, calculate healthcare facility–onset CDI rates at the organizational level and consider specifically calculating CDI rates by unit or ward (Table 3).
b. Provide CDI rates and CDI prevention process measures to key stakeholders including senior leadership, physicians, nursing staff, and other clinicians.
c. Provide the process and outcome measures to appropriate hospital staff and administrators on a regular basis as outlined in Section5: Performance measures.
8. Educate HCP, environmental service personnel, and hospital administration about CDI. (Quality of evidence: LOW)
a.Include risk factors, routes of transmission, local CDI epidemiology, patient outcomes, and treatment and prevention measures.
9. Educate patients and their families about CDI as appropriate. (Quality of evidence: LOW)
a.Although often not considered part of a program to reduce transmission of CDI and/or multidrug-resistant organisms, proper education may help to alleviate patient and family fears regarding being placed on contact precautions.
b. Include information about anticipated questions: general information about CDI, colonization versus infection, the hospital’s CDI prevention program, the components of and rationale for contact precautions, the risk of transmission to family and visitors while in the hospital and after discharge, and importance of hand hygiene by staff, patients, and visitors.
10. Measure compliance with CDC or WHO hand hygiene and contact precautions recommendations. (Quality of evidence: LOW)
a.Patient-to-patient transmission of *C. difficile* is thought to occur primarily through transient contamination of the hands of HCP with spores.
b. Glove use when caring for patients with CDI or touching surfaces in their rooms has been shown to be effective at preventing the transmission of *C. difficile*.
c. Hand hygiene practices in compliance with CDC or WHO guidelines may be important to *C. difficile* control and prevention.
Additional Approaches
1. Intensify the assessment of compliance with process measures. (Quality of evidence: LOW)
a.Contact precautions: gowns and gloves should be worn by all HCP who enter the rooms of patients on contact precautions.
b. Hand hygiene: hand hygiene should be performed at least on entry and exit from patient rooms. When hand washing is performed, determine whether proper technique is being used. If hand hygiene compliance or technique are not adequate, conduct interventions to improve hand hygiene compliance and technique.
c. Assess opportunities for improved antibiotic and/or diagnostic test utilization with improved compliance with and/or using additional antibiotic or diagnostic stewardship (see Section 4: Essential practices, parts 1 and 2).
2. As the preferred method, perform hand hygiene with soap and water following care of or interacting with the healthcare environment of a patient with CDI. (Quality of evidence: LOW)
a.When considering a CDI-specific hand hygiene measure, the priority should be to ensure adherence to donning gloves and proper technique when doffing to minimize the risk for self-contamination.
b. Ideally, after removing gloves, hand hygiene is performed before exiting the patient room when feasible.
c. Ensure proper hand hygiene technique when using soap and water.
d. Be aware that hand hygiene adherence may decrease when soap and water is the preferred method.
3. Place patients with diarrhea on contact precautions while *C. difficile* testing is pending. (Quality of evidence: LOW)
a.Place patients with new-onset, unexplained diarrhea on contact precautions when diarrhea is recognized. Employ measures (see Section 4: Essential practices, part 3), particularly use of gowns/gloves and disinfection of shared medical equipment. Contact precautions should be initiated as soon as diarrhea symptoms are recognized because this is the period of greatest *C. difficile* shedding and contamination.
b. Availability of private rooms or ability to cohort patients in nonprivate rooms before a CDI diagnosis may be a challenge for some hospitals. In these settings, the decision to place a patient on contact precautions in a private or cohort room while testing is pending can be based on several factors, including likelihood that patient will transmit *C. difficile*, turnaround time of CDI test results, and impact of contact precautions on hospital bed management.
c. If *C. difficile* testing is negative, and another infectious etiology that requires contact precautions is not suspected, contact precautions can be discontinued based on test type and clinical suspicion for CDI.
4. Prolong the duration of contact precautions after the patient becomes asymptomatic until hospital discharge. (Quality of Evidence: LOW)
a.CDC currently recommends contact precautions for patients with CDI for at least 48 hours after diarrhea resolves. However, some hospitals may choose to extend contact precautions for the duration of hospitalization even if symptoms have resolved.
b. Facilities must balance potential reduction in *C. difficile* transmission with individual patient risk of isolation related to contact precautions, which may include falls and socioemotional stress that can lead to symptoms such as behavior changes, anxiety, depression, and anger.
5. Use an EPA-approved sporicidal disinfectant, such as diluted (1:10) sodium hypochlorite, for environmental cleaning/disinfection. Implement a system to coordinate with environmental services if it is determined that sodium hypochlorite is needed for environmental disinfection. (Quality of evidence: LOW)
a.Sporicidal disinfectants registered with the EPA, including sodium hypochlorite, can be found in the EPA List K.
b. Data have not been consistent regarding the ability of sporicidal disinfectants, including diluted sodium hypochlorite, to control CDI through environmental decontamination.
c. When an EPA-approved sporicidal disinfectant is instituted for environmental decontamination, it is necessary to coordinate activities with environmental services.
d. When diluted (1:10) sodium hypochlorite is used, it is important to address several issues including measures to avoid toxicity to patients and staff, removal of organic matter from surfaces before use, and using freshly diluted or appropriately stored diluted sodium hypochlorite.
e. When a sporicidal method will be used only in rooms of patients with CDI, a system will need to be created to identify these patients to environmental service staff.
Unresolved Issues
1. Identification of asymptomatic carriers of toxigenic *C. difficile* using rectal or perirectal swabs and NAAT testing and placing those who are positive on contact precautions.
2. Implementation of touchless disinfection technologies.
3. Use of probiotics as primary prophylaxis.
4. CDI antibiotic prophylaxis for certain very high-risk patients who are receiving systemic antibiotics.
5. Use of gowns and gloves by family members and other visitors.
6. Use of admission-based alert systems that notify infection preventionists and clinical personnel about readmitted or transferred patients with a history of CDI.
7. Ongoing assessment of CDI knowledge and intensified CDI education among HCP.
8. Restriction of gastric acid suppressants.

Note. NAAT, nucleic acid amplification test; CDC, Centers for Disease Control and Prevention; WHO, World Health Organization; EPA, US Environmental Protection Agency; HCP, healthcare personnel.

**Table 2.** Quality of Evidence^a^

Level	Description
High	Highly confident that the true effect lies close to that of the estimated size and direction of the effect. Evidence is rated as “High” quality when there are a wide range of studies with no major limitations, there is little variation between studies, and the summary estimate has a narrow confidence interval.
Moderate	The true effect is likely to be close to the estimated size and direction of the effect, but there is a possibility that it is substantially different. Evidence is rated as “moderate” quality when there are only a few studies and some have limitations but not major flaws, there is some variation between studies, or the confidence interval of the summary estimate is wide.
Low	The true effect may be substantially different from the estimated size and direction of the effect. Evidence is rated as “Low” quality when supporting studies have major flaws, there is important variation between studies, the confidence interval of the summary estimate is very wide, or there are no rigorous studies.

a Based on the CDC Healthcare Infection Control Practices Advisory Committee (HICPAC) “Update to the Centers for Disease Control and Prevention and the Healthcare Infection Control Practices Advisory Committee Recommendations Categorization Scheme for Infection Control and Prevention Guideline Recommendations” (October 2019), the Grades of Recommendation, Assessment, Development, and Evaluation (GRADE)^166^ and the Canadian Task Force on Preventive Health Care.^167^

**Table 3.** Commonly Used *Clostridioides difficile* Infection (CDI) Surveillance Definitions ^8,56^

Case Type	Definition
Healthcare facility–onset CDI (HO-CDI)	CDI symptom onset ≥4 days after admission to an HCF, with day of admission being day 1.^a^
Healthcare facility–onset, treated CDI (HT-CDI) The proposed definition is currently being evaluated (see Section 2: Surveillance definitions for CDI, part 1e).	CDI symptom onset ≥4 days after admission to a healthcare facility (HCF), with day of admission being day 1, and ≥5 days of CDI treatment started within 2 calendar days of the positive *C. difficile* test; if a patient is discharged or transferred before receiving 5 days of treatment, any treatment will count.^a^
Community-onset, healthcare facility-associated CDI (CO-HCFA-CDI)	CDI symptom onset in the community or <4 days from admission (day of admission being day 1), provided that symptom onset was <4 weeks after the last discharge from an HCF, according to NHSN definitions.^56,a,b^
Indeterminate onset CDI	CDI case patient who does not fit any of the above criteria for an exposure setting, eg, CDI symptom onset in the community or <4 days from admission (day of admission being day 1) provided that symptom onset was >4 weeks but <12 weeks after the last discharge from an HCF.^a^
Community-associated CDI (CA-CDI)	CDI symptom onset in the community or < 4 days from admission (day of admission being day 1), provided that symptom onset was >12 weeks after the last discharge from an HCF.^a^
Healthcare-associated CDI (HA-CDI)	Includes cases of HO-CDI, CO-HCFA-CDI, and indeterminate per CDC Emerging Infections Program definitions.^8^
Community-onset CDI (CO-CDI)	Includes both CA-CDI and indeterminate CDI (distinct from CO-HCFA-CDI) per NHSN definitions.^56^
Unknown	Exposure setting cannot be determined because of lack of available data.
Recurrent CDI	A CDI episode that occurs 8 weeks (56 days) or less after the onset of a previous CDI episode, provided that CDI symptoms from the earlier episode resolved.

Note. HCF, healthcare facility; NHSN, National Healthcare Safety Network.

a When utilizing laboratory-based reporting symptoms, date and time of stool specimen collection can be used as a surrogate for symptom onset. If data on time a patient was admitted (in addition to date) and/or time stool was collected for testing are not available, CDI can be considered healthcare facility onset if stool is positive for toxigenic *C. difficile* or toxin after the third calendar day from hospital admission, where the first day is the day of admission (ie, a patient admitted on Monday with stool first positive for *C. difficile* toxin on Thursday or later is considered to have healthcare facility-onset CDI).

b CDC Emerging Infections Program definitions include CO-HCFA-CDI cases as defined above and indeterminate onset cases as defined below (ie, all CDI occurring <12 weeks after last discharge) in their specific CO-HCFA-CDI definition.^8^

**Table 4.** *Clostridioides difficile* infection (CDI) Prevention Process and Outcome Measures

Process Measures^a^	
Compliance with hand hygiene guidelines: *If hand hygiene with soap and water is the preferred method of hand hygiene when caring for patients with CDI, also assess proper hand washing technique with the same formula*.	(No. of observed proper hand hygiene episodes performed by HCP ÷ total no. of observed opportunities for hand hygiene) × 100 = % compliance with hand hygiene compliance
Compliance with contact precautions	(No. of observed patient care episodes in which contact precautions are appropriately implemented ÷ the no. of observed patient care episodes in which contact precautions are indicated) × 100 = % compliance with contact precautions
Compliance with environmental cleaning and disinfection	One specific measure of compliance for use in all hospitals cannot be recommended. However, many hospitals use checklists, environmental rounds, fluorescent markers, and/or ATP bioluminescence to assess the cleaning and disinfection process and cleanliness of equipment and the environment (see Section 4: Essential practices, part 5).
Outcome Measures^b^	
• Calculate CDI rates. • See Table 3 for case definitions.	
(No. of CDI cases in the population being monitored ÷ total number of patient days in the population being monitored) × 10,000 = No. of CDI cases per 10,000 patient days	

Note. HCP, healthcare personnel.

a Ongoing measurement of recommended CDI prevention practices to permit risk assessment of CDI.

b Ongoing measurement of incidence density of CDI for longitudinal assessment of outcomes related to the processes of care.
